# Content, Quality, and Assessment Tools of Physician-Rating Websites in 12 Countries: Quantitative Analysis

**DOI:** 10.2196/jmir.9105

**Published:** 2018-06-14

**Authors:** Fabia Rothenfluh, Peter J Schulz

**Affiliations:** ^1^ Institute of Communication and Health Università della Svizzera italiana Lugano Switzerland

**Keywords:** physician rating websites, content analysis, website quality, patient Web portals, rating tools, health information, health care quality assessment, patient reviews

## Abstract

**Background:**

Websites on which users can rate their physician are becoming increasingly popular, but little is known about the website quality, the information content, and the tools they offer users to assess physicians. This study assesses these aspects on physician-rating websites in German- and English-speaking countries.

**Objective:**

The objective of this study was to collect information on websites with a physician rating or review tool in 12 countries in terms of metadata, website quality (transparency, privacy and freedom of speech of physicians and patients, check mechanisms for appropriateness and accuracy of reviews, and ease of page navigation), professional information about the physician, rating scales and tools, as well as traffic rank.

**Methods:**

A systematic Web search based on a set of predefined keywords was conducted on Google, Bing, and Yahoo in August 2016. A final sample of 143 physician-rating websites was analyzed and coded for metadata, quality, information content, and the physician-rating tools.

**Results:**

The majority of websites were registered in the United States (40/143) or Germany (25/143). The vast majority were commercially owned (120/143, 83.9%), and 69.9% (100/143) displayed some form of physician advertisement. Overall, information content (mean 9.95/25) as well as quality were low (mean 18.67/47). Websites registered in the United Kingdom obtained the highest quality scores (mean 26.50/47), followed by Australian websites (mean 21.50/47). In terms of rating tools, physician-rating websites were most frequently asking users to score overall performance, punctuality, or wait time in practice.

**Conclusions:**

This study evidences that websites that provide physician rating should improve and communicate their quality standards, especially in terms of physician and user protection, as well as transparency. In addition, given that quality standards on physician-rating websites are low overall, the development of transparent guidelines is required. Furthermore, attention should be paid to the financial goals that the majority of physician-rating websites, especially the ones that are commercially owned, pursue.

## Introduction

### Background

The internet has become an invaluable resource for any kind of question or query one may search an answer for. The search and selection of a physician via the internet is no exception, especially if patient opinions can be easily obtained via the World Wide Web [1,2]. Physician-rating websites (PRWs) show numeric scores and textual appraisals about former patients’ encounters and experiences with a physician. However, not only do specialized websites for physician assessments offer user reviews of doctors but general commercial webpages such as Yelp also ask users to review medical professionals [3]. Yet the quality of PRWs and the rating tools they present to their users are largely unknown.

The content and quality of PRWs is a concern for both medical practitioners and website users. The former are afraid of unjustified reviews that do not reflect the true nature of their actual medical performance [4]. Biases in the user and the data, the risk of false allegations combined with website providers’ negligence to systematically control PRW reviews, the anonymity of the ratings, as well as health care consumers’ inability to judge certain aspects of care lead physicians to doubt the usefulness of PRW reviews [5]. Health care consumers on the other hand desire more quality of care information to improve their choices but have difficulties using such reports because of the complexity of the material [6,7]. To sum up, both physicians and health care consumers demand quality standards on PRWs that increase transparency while protecting both parties’ freedom of speech and privacy. These insights call for an assessment of the availability and quality of PRWs to evaluate to what extent physicians’ and health care consumers’ worries are justified.

### Study Objectives

A study assessing the quality, physician profile information, and rating tools present on PRWs across countries and languages has to our knowledge not yet been undertaken. This led us to the following research questions:

1. Of what website quality are PRWs? Which aspects of quality are most frequently met that are largely missing?
2. What information about the physicians and their practices is available on these websites?
3. How and based on which scales can users rate a doctor online?
4. How does quality and information content differ between countries?

## Methods

### Codebook

**Figure 1:**
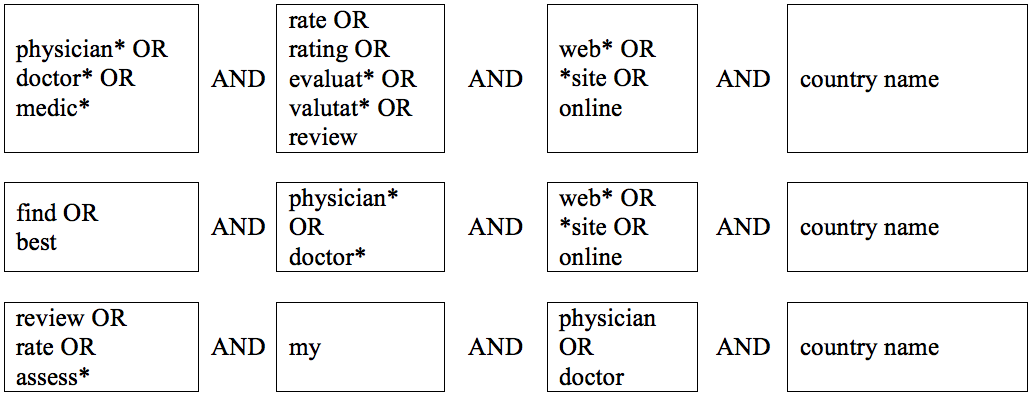
Search term strings entered in Google, Bing, and Yahoo to collect the website sample.

**Figure 2:**
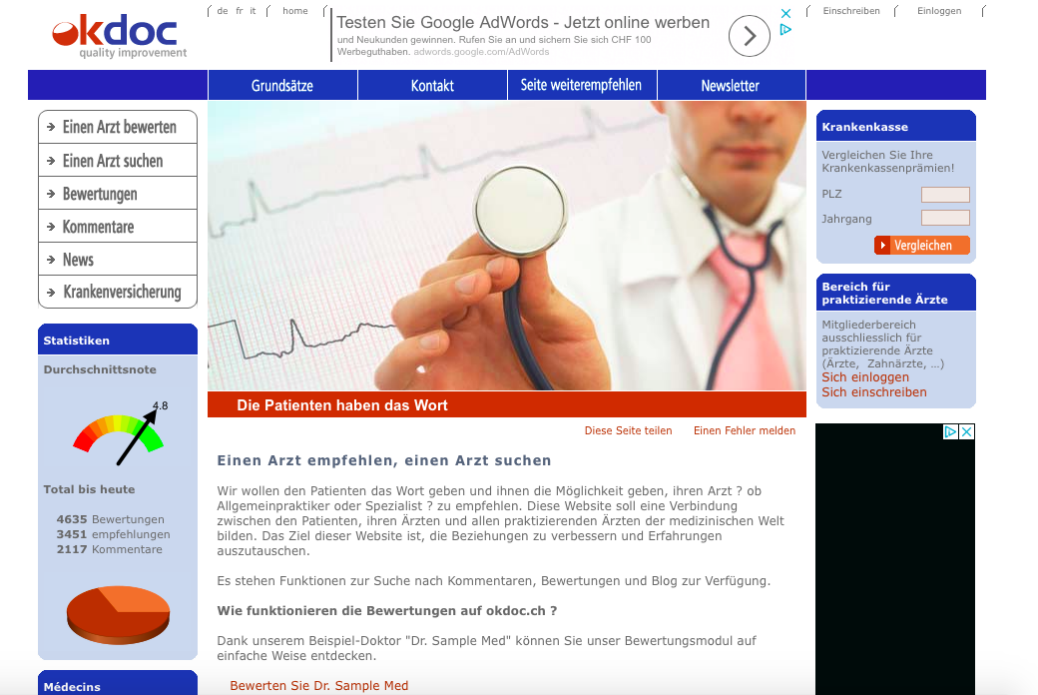
Screenshot of the physician-rating website okdoc.ch registered in Switzerland.

**Figure 3:**
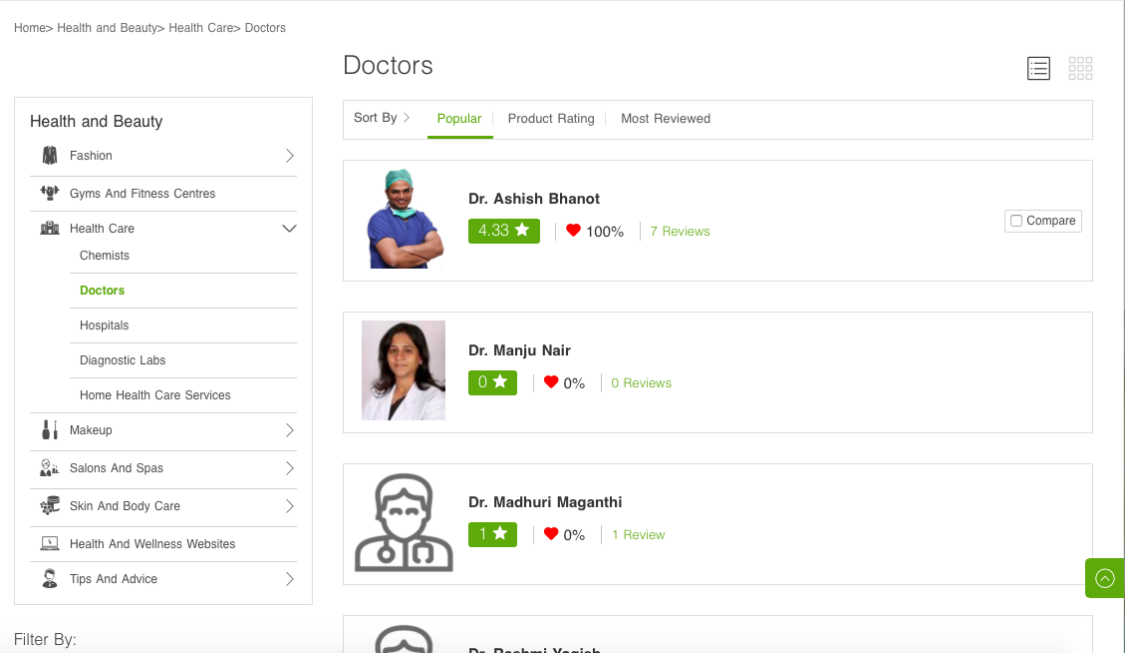
Screenshot of the physician-rating website mouthshut.com registered in India.

The website sample was collected through a Web search in August 2016 on the three largest search engines: Google, Bing, and Yahoo [8]. Web searches were conducted based on a list of keywords that were entered in English or German, based on the search country (Figure 1). Search engines were used with respective country codes (eg, in Germany we used search engine URLs ending in .de and in the United States URLs ending in .com) to mimic searches from residents looking for a doctor in their country. Websites were included in the sample if they fulfilled the following criteria: (1) accessibility in English or German, (2) retrievability via Web searches including one of the 12 preselected countries (United Kingdom, United States, Canada, Australia, New Zealand, India, Singapore, United Arab Emirates [Dubai], South Africa, Switzerland, Germany, and Austria), and (3) presence of ratings, evaluations, or written feedback sections to assess or rate physicians. We included PRWs from the United States and Germany in the study because the vast majority of publications up to date covered these two countries. To enlarge the sample, we included other countries where English or German is spoken. The first 100 webpages for each search term and engine were screened for the inclusion criteria, yielding a sample of N=208. The websites were coded from September 2016 to December 2016 and webcached. Examples of coded websites can be viewed in Figures 2 and 3 [9,10].

### Metadata

The first part of our coding consisted of metadata, namely about the owner of the website, registration country, coverage area, or upgrade features. These indicators were coded for presence and absence and summarized in a table.

### Website Quality

The second section of the coding focused on website quality. On the basis of literature [4,5,11-13], five quality dimensions for PRWs quality were developed and indicators accordingly derived. According to the definition applied in this study, a high quality PRW (1) publishes transparent, accurate, and neutral content from evident sources (eg, clear separation of advertisement and content); (2) respects the privacy and freedom of speech of both physician (eg, informs physicians about new ratings and asks physicians to reply to reviews) and health care consumers (eg, publishes anonymous reviews and verifies the identity of reviewers); (3) has check mechanisms in place to ensure the accuracy and appropriateness of information content reviews (eg, number of reviews that a health care consumer can write is limited and all reviews are checked before publication); and (4) is easy to use and navigate (eg, has filters present and search masks available). The breakdown of these dimensions led to 47 indicators, which can be found in Multimedia Appendix 1. The indicators were not weighted because the opposing views about the weight of individual indicators from physicians’ and patients’ perspectives can hardly be reconciled (eg, although physicians oppose anonymous reviews, patients highly value them as they fret about the impact of a negative review on future care).

### Professional Information

The third section on information content consisted of 25 items covering information on the professional and educational background of the physician, practice access, and contact information, as well as personal data about the doctor. These indicators were developed iteratively; first, indicators were derived from a scoping review by Victoor and colleagues [14] and a study by Rothenfluh and Schulz [15] on aspects that were cited to be for patients when choosing a physician. In a second step, during the pretest, more information items were added until saturation was reached. All items that appeared during the actual coding and did not fit into the indicators collected during the development and pretest were ordered into separate categories labeled *other*.

### Scales or Rating Tools

A fourth section of the codebook was dedicated to the scales or rating tools available on PRWs to assess a physician. An earlier study by Rothenfluh and Schulz [15] identified indicators that are, according to physicians and health care consumers, important to identify a good physician and assessable by patients. These indicators were developed based on Donabedian’s quality of care model [16,17] that subdivides care quality into dimensions of structure (infrastructure, staff, equipment, organization, and accessibility), process (technical and interpersonal skill of the physician), and outcome (results of the treatment) of health care. All rating tools on the websites in this study’s sample were coded based on this structure.

### Website Traffic Rank

Furthermore, the website traffic rank, an indicator for website popularity, was recorded for each webpage on January 26, 2017, based on the Alexa Global Traffic Ranking [18]. For each website, both the traffic rank globally as well as the national ranking were recorded.

### Coding Procedure

The codebook was pretested twice based on 10 websites each. Adjustments were made where necessary. To assure the reliability of the data, a second coder was trained based on the codebook (three sessions of 3 hours). The level of agreement between the first and second coder was compared after each session and differences were discussed. This process was repeated three times until sufficient agreement was reached. Then, the second coder independently coded a randomly drawn subsample of 29 websites (20%), which is sufficient according to Riffe and colleagues [19].

## Results

### Coding Procedure and Intercoder Reliability

On the basis of our Web search, the initial sample of websites consisted of 208 websites, which was consolidated to 143 during the coding process because of temporary inaccessibility of websites, deletion or cessation, or the disappearance of an online review function. The intercoder reliability based on Krippendorff alpha [20] was satisfactory (average over all items alpha=.95) after five items were excluded because of agreement below alpha=.667, which is the lowest cutoff according to Krippendorff [20,21]. Among the excluded items were the completeness of the presented physician profiles and the format and source in which the profile information was presented (eg, open-ended text sections filled in by the physician, information provided by the provider, or not identifiable).

### Metadata

In terms of metadata (Table 1), we found that the vast majority of websites were operated by commercial for-profit companies (120/143, 83.9%). Business models included various profile upgrade options for physicians that are often payable through monthly or annual fees (see detailed features in Table 1). Such benefits included that physicians could pay for commercial services (42/143, 29.4%) such as online appointment booking, or to enter the biddings to offer a client treatments (especially in dentistry). Furthermore, doctors could pay for their profiles to be further up or listed first on users’ search results (51/143, 35.7%).

### Website Quality

A quality index of 47 items was calculated (see Multimedia Appendix 1), awarding one point per quality criterion fulfilled, yielding scores between 0 and 47. The mean quality score was 18.67 (SD 4.13), ranging from 8 to 29 points, with 69.3% (99/143) of the sample scoring between 15 and 22 points. Overall, the three highest individual website quality scores of the 143 coded websites were attained by one website registered in Germany, one in Austria, and one in the United Kingdom. The individual websites with the lowest website quality were a website registered in Singapore and one in Canada reaching only 8 points or 9 points, respectively.

**Table 1:** Website metadata and features offered (N=143).

Metadata			n (%)
**Owner of the website**			
	Unknown or not identifiable		11 (7.7)
	Commercial for-profit company		120 (83.9)
	Nonprofit organization		5 (3.5)
	Medical professional organization		1 (0.7)
	Political institution or governmental organization		2 (1.4)
	Other (eg, hospital and health insurance)		4 (2.8)
**Physician information updates and upgrades offered**			
	No upgrades offered		11 (7.7)
	Profile update offered, no indication if at a cost		27 (18.9)
	Profile update offered for free		4 (2.8)
	Profile update offered at a cost		12 (8.4)
	Profile update offered for free, upgrades available at a cost		84 (58.7)
	No information available		5 (3.5)
**Types of cost billing**			
	No information available		33 (23.1)
	Absent		56 (39.2)
	Fee (weekly, monthly, or annual)		41 (28.7)
	Billing by case (per client served or gained)		5 (3.5)
	Fee (monthly, annual) plus billing by case		7 (4.9)
	Cost per information item the doctor adds		1 (0.7)
**Upgrade benefits offered for free and at a cost**			
	**Better or higher listing position of physician profile**		
		For free	7 (4.9)
		At a cost	51 (35.7)
	**Seal that the doctor is excellent**		
		For free	3 (2.1)
		At a cost	11 (7.7)
	**Google indexing for higher position in search results**		
		For free	3 (2.1)
		At a cost	19 (13.3)
	**Costumer service or profile maintenance**		
		For free	5 (3.5)
		At a cost	22 (15.4)
	**Profile presentation enhancement by adding pictures, videos, or more information about the doctor**		
		For free	14 (9.8)
		At a cost	61 (42.7)
	**Physicians can respond to patients’ reviews**		
		For free	22 (15.4)
		At a cost	10 (7.0)
	**Commercial benefits (eg, online appointment scheduling and bidding system for treatments)**		
		For free	11 (7.7)
		At a cost	42 (29.4)

**Table 2:** Information available on doctors’ profile pages about the physician and practice (N=143).

Available information on physicians’ profiles	Present, n (%)
Address of the practice	136 (94.4)
Phone number of the practice	118 (81.9)
Directions to find address	111 (77.1)
Detailed degree or specializations of the doctor	93 (64.6)
List of medical conditions that the physician treats	85 (59.4)
List of medical procedures (treatments, etc) offered	86 (59.7)
Website of the physician and practice	78 (54.2)
Office hours of the practice	75 (52.1)
Doctor’s years of work experience	60 (41.7)
Languages the physician speaks	58 (40.3)
Insurance plan restrictions information (eg, if a physician accepts only private insurance or self-payment)	58 (40.3)
Training and degrees of the doctor	70 (48.6)
Email address of the practice	67 (46.5)
List of medical continuous education courses the physician completed	56 (38.9)
Gender of the physician	49 (34.3)
Awards and honors that the physician received	34 (23.6)
Scientific publications by the physician	28 (19.4)
Insurance plans or health insurance companies the provider works with	26 (18.1)
Practice access information for handicapped people	21 (14.6)
Doctor’s memberships in physician associations	15 (10.4)
Age of the physician	12 (8.3)
Physician’s external quality assessment results	10 (6.9)
Number of surgical procedures that the physician performed in his career (surgeon volume)	4 (2.8)
Legal actions after errors, malpractice, or sanctions that were filed against the physician	2 (1.4)
Personal information (eg, doctor’s marital status and family information)	2 (1.4)

Overall, indicators related to transparency, such as the type of website provider (132/143, 92.3%) and website background information (eg, website owner [122/143, 85.3%] or terms and conditions [128/143, 89.5%]) were available on the vast majority of websites. Furthermore, basic quality assurance criteria such as limiting the number of reviews by the same user were present in almost three-quarters of websites. However, the assurance of transparency proved to be less common when related to financial benefits for the website provider. For example, 75.5% (108/143) of websites did not clearly separate advertisement from content, and more than 69.9% (100/143) had some form of physician ad present. Furthermore, HON code certifications, a label that marks trustworthy health and medical information [22], were only displayed on 5.6% (8/143) of the cases. Statistical quality assurance indicators such as a minimum number of reviews online before reviews are displayed were largely absent (only in 9.3%, 13/143 present). Remarkably, only 4.2% (6/143) of the websites stated that they notify the physicians whose profile goes online. This makes keeping track of their potentially numerous online profiles difficult for physicians. Furthermore, merely 11.9% (17/143) provided the physician’s medical board registration number, which can be an indicator for users that may help him identify physicians who passed the country’s requirements to practice medicine.

### Professional Information

When Web users search for a doctor online, the amount and kind of information they find about the doctor and his or her practice may help users’ decide whether to consult a certain doctor or not. Therefore, information content was added up to a score between 0 and 25 (see indicators in Table 2). With a mean of 9.95 (SD 4.01), the vast majority of websites had little information available about the doctors listed. The information most commonly available about doctors were the address of the practice (136/143, 94.4%), the practice phone number (118/143, 81.9%), and directions to find the practice (111/143, 77.1%), which resembles the information one could also retrieve from a phone book.

### Quality and Information Content on Physician-Rating Websites in Different Countries

To shed light on potential differences between countries’ PRW information content and quality, the websites were split based on their registration country. According to our quality measure (0-47 points), websites registered in the United Kingdom had the best quality PRWs (mean 26.50, SD 1.00), followed by Australia (mean 21.50, SD 0.71) and Germany (mean 20.72, SD 4.12). It has to be noted though that both the United Kingdom and Australia had few websites registered in their countries and low variability compared with, for example, Germany, where the two highest quality websites were registered (see Table 3). In terms of information content (score between 0-25), websites registered in Australian scored the highest (mean 15.50, SD 6.64), followed by German ones (mean 10.00, SD 2.48).

Numerous websites (35/143) had multinational coverage areas, which may or may not overlap with the registration country according to which the countries are listed in Table 3. For example, Canada had only 3 websites registered, but 10 pages covered physicians practicing in Canada. The United States on the other hand had 40 websites registered there, but only 28 exclusively displayed physician profiles from doctors practicing in the United States. Therefore, a comparison of websites across countries should be interpreted with caution. It should further be noted that the quality scores of websites not registered in one of our sample countries had the lowest quality, which points to potential legal issues that may emerge based on this gap between registration country and coverage area.

### Scales or Rating Tools

PRWs may invite users to score doctors based on numeric scales, ask for written feedback, or a combination of both. In our sample, 15.3% (22/143) of the websites asked for numeric physician assessments only, 4.2% (6/143) for written reviews exclusively, and 76.9% (110/143) provided the option to give feedback based on both predefined rating scales as well as to type reviews or testimonials. Most frequently, PRW users were invited to rate the overall treatment encounter (75/143, 52.1%), punctuality and wait time in practice (51/143, 35.7%) or for the next appointment (27/143, 18.9%), and whether the user would recommend that specific doctor (44/143, 30.8%). Furthermore, users may be asked to rate the office environment (eg, practice comfort and cleanliness: 33/143, 23.1%), or the friendliness and courteousness of the staff (32/143, 22.4%). Looking at the assessment tools in terms of broader dimensions, one or several indicators on interpersonal aspects of care could be assessed on 65 (45.5%) of the coded websites. Specifically, information provision by the physician (comprehensiveness, clarity, questions answered, etc), bedside manner, helpfulness, and empathy (25/143, 17.4%), or if the doctor spent sufficient time with the patient (17/143, 11.8%) could be rated. Yet, scales on which users were asked to score one or several technical aspects of care were less frequently present (37/143, 25.9%). For example, few websites asked users to evaluate the physician’s knowledge (16/143, 11.2%), competence (9/143, 6.3%), or the correctness of the diagnosis (9/143, 6.3%). Users were inquired to rate aspects concerning one or several rating items on the outcome of care on 24 (16.8%) of the websites. Assessment items included, for example, the presence and quality of the follow-up (9/143, 6.3%) or the efficiency of the treatment (8/143, 5.6%). Further rating scale items can be found in Table 4.

**Table 3:** Website quality and information by registration country in descending order of quality.

Registration country^a^	Number of websites	Website quality (summative score 0-47)			Information content (summative score 0-25)		
	N	Mean (SD)	Minimum	Maximum	Mean (SD)	Minimum	Maximum
United Kingdom	4	26.50 (1.00)	26	28	8.00 (6.64)	2	16
Australia	2	21.50 (0.71)	21	22	15.50 (2.12)	14	17
Germany	25	20.72 (4.12)	15	29	10.00 (2.48)	5	16
Austria	13	19.85 (3.60)	14	28	12.85 (4.00)	8	20
Switzerland	11	18.91 (4.04)	13	25	9.27 (2.76)	3	13
United States	40	18.25 (3.03)	12	24	9.80 (4.24)	0	20
Singapore	5	18.20 (5.85)	8	22	10.40 (5.23)	6	17
South Africa	6	18.17 (3.43)	14	23	9.33 (3.93)	5	14
India	14	17.79 (1.81)	15	23	9.36 (4.52)	1	15
United Arab Emirates (Dubai)	3	17.00 (2.00)	15	19	13.33 (4.73)	8	17
Canada	3	15.67 (7.23)	11	24	8.33 (2.89)	5	10
Other or not identifiable registration country (eg, Spain and Romania)	17	15.29 (4.66)	9	22	8.53 (3.81)	3	16

^a^The registration country according to which websites are listed here is not always equivalent with the coverage areas of these websites.

**Table 4:** Rating scale items present on physician rating websites.

Dimension and indicators			Present, n (%)
**Structure**			
	**Infrastructure**		
		Office environment, cleanliness, comfort	33 (23.1)
		Instruments in the practice to make the diagnosis or execute the treatment	9 (6.3)
		Reachability of the practice by car or public transport	8 (5.6)
	**Organization**		
		Punctuality, wait time in practice	51 (35.7)
		Scheduling or making appointments	23 (16.1)
		Waiting time until the next appointment	27 (18.9)
		Reachability of the practice via phone	9 (6.3)
		Notification of patients in case of appointment delays or cancellations	3 (2.1)
		Teamwork between physician and his team	2 (1.4)
		Number of staff present in the practice to welcome and take care of patients	1 (0.7)
	**Staff**		
		Staff friendliness and courteousness	32 (22.4)
		Staff experience and training	5 (3.5)
**Process**			
	**Interpersonal**		
		Comprehensiveness and completeness of information provision	31 (21.7)
		Social skills of the doctor (attentiveness, helpfulness, empathy)	25 (17.5)
		Amount of time spent with the patient	17 (11.9)
		Friendliness of the physician	16 (11.2)
		Physician’s (active) listening skills	15 (10.5)
		Conversation climate with the doctor	15 (10.5)
		Trust in physician	13 (9.1)
		Confidentiality, protection of privacy	6 (4.2)
		Information provision about how to handle the illness or disease	10 (7.0)
		Shared decision about the course of action together with the patient or shared decision making	6 (4.2)
		Doctor’s effort to engage the patient in shared decision making	6 (4.2)
		Physician’s skill to assess the patient’s handicaps and presentation with appropriate information and treatment options	1 (0.7)
		Communication and narration during the treatment execution	1 (0.7)
	**Technical or medical**		
		Physician’s knowledge	16 (11.2)
		Physician’s competence	9 (6.3)
		Correctness of the diagnosis, diagnostic ability of the physician	9 (6.3)
		Improvement of the patient’s health status	8 (5.6)
		Timely referral to a specialist or the hospital if needed	5 (3.5)
		Completeness and quality of anamnesis	4 (2.8)
		Quality and variety of treatment suggestions	3 (2.1)
		Cost consciousness of the physician when making tests or giving out medications	3 (2.1)
		Physician’s experience	2 (1.4)
		Responsible medication prescription	2 (1.4)
		Systematic proceeding of physician to reach the correct diagnosis	2 (1.4)
		Timeliness or promptness of the diagnosis and initiation of the treatment	2 (1.4)
		Correctness of treatment execution by the physician and his team	1 (0.7)
		Quality of the information provided to the patient	19 (0.7)
		Physician’s competence to execute the treatment competently	1 (0.7)
**Outcome**			
	Likelihood of recommendation		44 (30.8)
	Satisfaction with the doctor		12 (8.4)
	Presence and quality of the follow-up care		9 (6.3)
	Efficiency of the treatment or cost-benefit ratio		8 (5.6)
	Price of the treatment		4 (2.8)
	Cost coverage by the health insurance		2 (1.4)
	Patient’s increase in knowledge about his disease or injury		1 (0.7)
	Number or kind of complications^a^		0 (0.0)
	Patient loyalty or patient’s intention to return for future or follow-up treatments^a^		0 (0.0)
**Summative and other**			
	Summative or overall score		75 (52.4)
	Other organization scores		14 (9.8)
	Other interpersonal scores		12 (8.4)
	Other overall scores		2 (1.4)
	Other technical scores		2 (1.4)

^a^These indicators emerged in the literature as important to identify a good doctor but were not present on any physician-rating websites.

### Website Traffic Rank

The website traffic rank on Alexa was recorded on January 26, 2017, serving as an indicator of the popularity of the PRWs in this study in their registration country. First, it should be noted that 8 websites did not have an Alexa global rank [18], while 44 were not ranked locally. The most frequently visited website was Yelp on a global scale, ranked on position 282, followed by Web MD ranked number 501 and Yellow Pages on position 1634 worldwide. Given that Yelp and Yellow Pages are primarily directories, they attract most likely the vast majority of their traffic through webpage visits unrelated to physician searches or review writing. The website most popular within a country was Herold in Austria on rank 86 nationally, followed by Just Dial in India positioned on rank 63, and by NHS Choices in Great Britain on national rank 143. Herold as well as Just Dial are also first and foremost directories, likely attracting most of their traffic through address searches, whereas users may not even be aware of its function to rate doctors. NHS Choices on the other hand is Britain’s public health care system’s webpage and therefore the first point of entry or first address about health issues in the United Kingdom.

## Discussion

### Principal Findings

This study assessed the quality, information content, and rating tools on websites providing physician rating in 12 countries of German or English language. Most websites were registered in the United States and Germany. Yet, one has to differentiate between registration country and coverage area of those websites as this has important legal implications for the physicians listed. On average, quality and information content of PRWs in various countries differed tremendously, whereas the quality of the majority did not even achieve half of the maximum quality points possible.

To our knowledge, this is the first study that analyzed the quality, physician information content, and rating tools of doctor rating websites in 12 countries on a broad basis. Prior content analyses that focused on the structure and content on PRWs were more restrictive in their inclusion criteria only coding websites that exclusively displayed physicians, leading to smaller sample sizes of 8 [23] or 28 websites, respectively [24]. Due to our broader inclusion criteria (see Methods section), we analyzed a more heterogeneous sample, including all websites that had some form of physician rating or review present, consequently providing a broader picture.

The majority of the websites in our sample was commercially owned (120/143, 83.9%). On such profit-focused websites, revenue is often generated via upgrades of physician profiles. This can be beneficial for both patients and physicians if features such as online appointment bookings are offered. However, other upgrades such as purchased top listing positions, which were present on 35.7% (51/143) of the websites, are problematic as they are often not evident as such to the user. As known from research in marketing, because of primacy or position effects, people tend to choose the first option on a list (eg, [25-27]). However, a purchased top physician listing may not reflect the actual quality of the doctor, thereby potentially misleading users.

Overall, the quality of the websites in our sample was mediocre. The vast majority attained less than half of the maximum score (between 15-22 points), whereas the highest quality website attained 29 out of 47 points. Additional information such as surgeon volume [28] or physician notification when the profile goes online were absent in almost 95% (6/143) of all websites. Furthermore, only a quarter of websites contained physicians’ replies to reviews, even though health care consumers report physician feedback to be crucial [13].

New and transparent quality guidelines are called for. Such quality guidelines should strengthen the rights of both physicians and health care consumers. In Germany, such guidelines have been developed. However, as the results of this study show, this did not necessarily translate into higher quality PRWs overall [11]. Independent, nonprofit companies such as the HON code society [22] for the quality of health information have paved the way toward globally recognized labels. For the development of a PRW quality label, a mixed committee of patients and physicians should be involved, so that both patients’ and doctors’ wishes and concerns find their perspectives and needs represented in the development of such a label. Given the diversity of medical specializations (ie, general practitioners may require a different skill set than neurosurgeons) and the knowledge base of patient (ie, a very informed long-term diabetes patient may have a different skill to assess a doctor than a patient who visits a doctor for the first time in 20 years to find out that he is suffering from diabetes), PRWs should pay tribute to such differences.

In terms of what users are asked to rate about their doctors, we found that overall scores or aspects of the organization, such as waiting time, appeared most frequently. Outcome of care and technical aspects of the physician are less often listed rating tools. Furthermore, rating websites that asked users to assess structural or interpersonal aspects of their care ranked higher on website quality than websites on which those rating scales were absent. This is in line with previous studies that report that overall scores, communication, and structural factors are the most frequently available doctor assessment tools [23,29]. Emmert and colleagues [23] further found that only a minority of PRWs asked users to assess process quality or treatment outcomes [23], which our study confirms.

A study by Rao and colleagues [30] reported that health care consumers failed to correctly judge technical quality of care aspects, whereas other studies suggest a positive association between PRW ratings and objective care measures [31], or Facebook recommendations and hospital readmissions [32]. Some studies suggest that there is a knowledge gap between doctors and patients related to medical knowledge [5], which makes it difficult for health care consumers to accurately evaluate a physician’s medical performance. A study by Rothenfluh and Schulz [15] reports further that physicians and health care consumers are indeed reflective of their own capabilities to assess certain care aspects, especially if highly technical. The findings from this study evidence, however, that only a minority of websites present technical or medical criteria (37/143, 25.9%) to be assessed by patients, which may be a reassuring finding for doctors. Hence, these findings can debilitate some ethical concerns raised in the PRW literature (see [4,5]).

Beyond numeric reviews, written patient testimonials should also be focused on more in this context. In our sample, patient narratives have been present on 81.1% (116/143) of the PRWs we analyzed. These narratives have been promoted as fruitful tools to obtain patient quality of care feedback, yet, it is advocated that they should be collected based on strict standards, showing promising results [33]. Our study revealed that only 26.6% (38/143) of the pages we analyzed provided instructions on how to provide meaningful and appropriate written testimonials. Furthermore, not even a third (30.8%, 44/143) of the websites in our sample systematically screened all patient reviews before they went online. This calls for action and enforcement of stricter quality guidelines on PRWs. Furthermore, given that numeric physician ratings are often not in line with written reviews, they can also cause contradictions within reviews [34]. Hence, further research is needed on how ratings, as well as narratives, could be more effectively elicited to provide meaningful and valuable feedback for physicians and insightful information for patients.

The quality of the websites differed between the 12 countries, with websites registered in the United Kingdom and Australia scoring highest. Yet, the registration country was not necessarily equivalent to the website’s coverage area. This could create difficulties, especially for doctors who want to take legal action against false or defamatory reviews. Even though there are the first publications on the legal grounds of PRWs, such as applying defamation law and medical nondisclosure agreements [12], the situation remains country-specific and complex. Newspaper articles on court cases in various countries, including Europe and North America, show that actions taken by physicians against defamatory comments are sometimes, but not always, successful [35-37]. A court decision in Germany shows though that the law increasingly recognizes the physicians’ perspectives on PRWs, especially when business interests compromise the neutrality of the displayed information. A ruling by the German High Court forces a PRW to delete doctors’ profiles when the doctors explicitly request for it. A PRW was sued because it displayed advertisements of upgraded doctor profiles on detail pages of physicians without such an upgrade, putting the doctors with a nonupgraded profile into a disadvantageous position [38]; Doctors could not request the removal of their profiles. The new ruling by the German High Court changes this situation. As a consequence, this ruling may change the landscape of physician profile upgrades in other countries as well, favoring transparency, while punishing practices that may distort users’ perception, such as profile upgrades.

Beyond a discussion and further research on legal issues on PRWs, country-specific differences in terms of the number, content, and quality of such websites may also be related to the health systems in which they operate. A health care system such as that of the United Kingdom is publicly financed and therefore leaves less choice to patients [39]. In countries such as the United States, on the contrary, individuals pay their health care expenses mostly out of their pocket [40]. Hence, PRWs in such systemically diverse countries may also lead to the emergence of dissimilar PRWs. Self-payers may therefore be more interested in comparisons of physicians, potentially explaining the large number of PRWs in the United States compared with, for example, the United Kingdom. However, these are just hypotheses, calling for further investigation.

The large number of websites that we found, for example, in Germany or the Unites States, point to a challenge doctors face; they have to stay on top of incoming reviews and to respond to them. Given the large number of review websites, this poses a daunting task, especially if websites do not inform doctors when their profiles or a review on them goes online. Only 14.0% (20/143) of the websites in our sample stated to inform doctors when a new review on them is posted, and 4.2% (6/143) notify physicians when their profile goes live. Given that physicians already have extraordinarily long working hours [41], replying to patient comments on such numerous websites in a timely manner can become burdensome, or even overwhelming [42]. It is therefore not surprising that only 34% of physicians surveyed in the United States reported to have made changes to their online profiles [43], even though the importance of responses to posted reviews is highly important for patients [13]. This situation has already opened up a new business opportunity: marketing firms offer physician profile maintenance at a cost [44-46]. This development is alarming because contrary to creating a physician-patient dialogue to improve quality of care [47] it outsources this potentially valuable feedback loop.

This study pointed to various shortcomings on websites that offer physician rating tools, reaching from low quality and limited information content to biased physician profile display. Nevertheless, online patient ratings of care quality can provide valuable and timely insights into shortcomings in care quality. Several studies have hinted at the association between objective care quality and patient feedback [32,48,49]. For example, a study by Glover and colleagues showed a significant association between Facebook ratings and hospital readmission rates, whereby lower ratings were associated with higher readmission rates [32]. Furthermore, patient ratings have the potential to change patients’ choice of doctors or hospitals, thereby encouraging doctors to adjust their practicing based on negative reviews [48]. This has been evidenced in a German study that showed how doctors who read their negative reviews and also responded to them, make an effort to improve the aspects that were criticized in their work [47]. Hence, the need for the public to be involved in quality of care reporting is increasingly suggested [49,50]. Yet, the assurance of quality and content on PRWs asks for further research and knowledge translation into practice.

Given that the vast majority of PRWs in our study was commercially owned, it remains questionable whether more neutral providers such as nonprofit organizations could outperform the existing websites in terms of traffic at the present state. As almost a third of websites sell profile upgrades or higher listing positions to physicians, a sign or label should clearly point out the absence of such business models. This can raise users’ awareness and incentivize commercial websites to change their business strategy. Furthermore, a label on PRWs could serve as quality assurance certification that helps both physicians and patients to better navigate toward ethical and high-quality physician-rating webpages. In addition, the number of reviews would have to be increased; the more reviews are present, the higher the statistical representativeness and the less subject single reviews are to outliers. As an outcome, reviews would be created in an environment safe for both parties, inspiring health care improvements and constructive dialogue, thereby ultimately raising transparency and quality in health care.

### Limitations

This study has to be considered under certain limitations. First, as the internet is a fast-changing environment, this content analysis of PRWs only provides a momentary picture of the situation in the 12 countries included in our sample. To respond to this issue, we provide readers with insight into the websites as they were when they were coded in the form of webcaching. Yet, webcached sites may not necessarily provide the same user experience.

Second, the Web search for PRWs only included pages that appeared based on the outlined search terms. The search terms we applied may not be complete though because of regional differences in search strings. Hence, some websites offering rating functions of physicians may not have been included in the sample.

Third, this content analysis was limited to information that was visible when users accessed the website (publicly available without user registration). However, it is possible that websites adhered to quality criteria in the version available to registered users, which we as unregistered visitors failed to notice. As a result, this study only attempts to draw conclusions based on publicly available data.

Fourth, the indicators comprising the website quality score were not weighted. Although it is undebated that some indicators are very important (eg, physicians can be high listing positions), other indicators are difficult to weight. For example, whether reviews should be published anonymously is debated among doctors and patients. Doctors value transparent publication of reviewers’ names and demand that when they are reviewed and rated, the authors of such reviews should reveal their identities as well. For patients on the other hand, it is crucial that their opinions remain anonymous as they fret about the impact of negative reviews on their future care encounters with the same or other providers. Hence, we opted to not weight the single indicators but to calculate a simple mean and to point the reader to the table where all indicators are listed separately. This way, the reader can draw his or her own conclusions.

### Conclusions

This study evidences that websites that provide physician rating should improve and communicate their quality standards, especially in terms of physician and user protection, as well as transparency. In addition, given that quality standards on PRWs are low overall, the development of transparent guidelines is required.

## Appendix

Multimedia Appendix 1 Quality criteria fulfillment levels.

## Abbreviations

PRW: physician-rating website
