# Supplementary material for: Content, Quality, and Assessment Tools of Physician-Rating Websites in 12 Countries: Quantitative Analysis
Source: J Med Internet Res. 2018 Jun 14;20(6):e212. doi: 10.2196/jmir.9105 (PMC6024097; doi:10.2196/jmir.9105)
Supplement: Multimedia Appendix 1 [file jmir_v20i6e212_app1.pdf]

Appendix 1. Quality criteria fulfillment levels.

**Quality dimension**		**Fulfilled, N (%)**
**Transparent, accurate, and neutral content from evident sources**		
	The type of website owner is identifiable	132 (92.3)
	Location search criteria (location, city, and community) are present	131 (91.6)
	The website's general terms and conditions are present	128 (89.5)
	The manager or owner of the website is listed	122 (85.3)
	The website transparently communicates from where the physician profile information was extracted	106 (74.1)
	Users need to register or provide information (personal or impersonal) to submit a review	103 (72)
	The email address of the website manager is listed	103 (72)
	It is clearly visible based on which criteria or rules physicians are listed in the search results page	58 (40.6)
	Review computation is transparent (more details about the computation process and the items building average scores can be viewed)	44 (30.8)
	There are no physician advertisements present	56 (39.2)
	The website does not sell upgrade profile enhancements to physicians	42 (29.4)
	The website transparently separates advertisements and website services or content	35 (24.5)
	Physicians' medical board registration number is listed	17 (11.9)
	The website states when a physician profile was last updated	16 (11.2)
	The website is HON Code certified (label present)	8 (5.6)

**Respects the freedom of speech and privacy of physician and health care consumer**		
	Reviews are anonymously or semi-anonymously published to protect the user	125 (87.4)
	Users are instructed on appropriate behavior on the physician-reported websites (PRWs)	82 (57.3)
	Physicians can request partial or full profile or information withdrawal	63 (44.1)
	User data is not transmitted to third parties	38 (26.6)
	Physicians can reply to patients' reviews	36 (25.2)
	All physician profile changes/ modifications are free for the listed physicians	31 (21.7)
	Cookies do not automatically collect data about the user	3 (2.1)
	User can identify if the physician's approved or claimed his or her own profile	14 (9.8)
	The rating website clearly states that reviews posted on that website are not shared with or forwarded to other platforms	7 (4.9)
	User information that was collected by the website is not processed or analyzed by the website provider	0 (0)
**Mechanisms to ensure accuracy and appropriateness of information content and reviews**		
	The website limits the number of evaluations by the same user	107 (74.8)
	A contact email address or button on the physician's profile page is available to report misuse of the website	106 (74.1)
	Physicians are protected in case users post defaming criticism	105 (73.4)
	The website has a feature to identify machine insertion of reviews	104 (72.7)

	User are asked to submit information about themselves before they can post a review	69 (48.3)
	Reviews are systematically checked by a website administrator before publication	44 (30.8)
	User instruction on appropriate behavior on the PRWs (present on the review page)	38 (26.6)
	Physicians are notified before a new post or review goes online	20 (14)
	Minimum number of reviews (more than one) required for profiles to go online	9 (6.3)
	Reviews expire after a certain time period	9 (6.3)
	There is a warning message present on the written review page indicating that reviews are subjective and should be viewed with care	8 (5.6)
	Physicians are notified when their profile goes online	6 (4.2
**Easy to use and navigate**		
	Search criteria “specialization” is present	133 (93)
	Search criteria “name of the physician” is present	100 (69.9)
	Providers close by are featured one click away from the profile the user is viewing	50 (35)
	Providers of the same specialization are featured are one click away from the profile the user is viewing	54 (33.7)
	There are filters available that help users limit physician choices based on their preferences	48 (33.6)
	Search criteria “patient’s medical condition” is present	41 (28.7)
	Search criteria “sociodemographic criteria” is present	16 (11.2)
	Search criteria “health insurances the physician accepts” is present	15 (10.5)
	Users can compare two or more doctors side by side	11 (7.7)
	The websites provides user aids for people with handicaps	1 (0.7)
